# Potential benefits of integrating ecological momentary assessment data into mHealth care systems

**DOI:** 10.1186/s13030-019-0160-5

**Published:** 2019-08-09

**Authors:** Jinhyuk Kim, David Marcusson-Clavertz, Kazuhiro Yoshiuchi, Joshua M. Smyth

**Affiliations:** 1 Department of Informatics, Shizuoka University, 3-5-1 Johoku, Naka-ku, Hamamatsu, Shizuoka, 432-8011 Japan; 2 Department of Biobehavioral Health, The Pennsylvania State University, University Park, PA USA; 3 Department of Psychology, Lund University, Lund, Sweden; 4 Department of Psychology, Ludwig Maximilian University of Munich, Munich, Germany; 5 Department of Stress Sciences and Psychosomatic Medicine, Graduate School of Medicine, The University of Tokyo, Tokyo, Japan; 6 Department of Medicine, Hershey Medical Center and The Pennsylvania State University, Hershey, PA USA

**Keywords:** Mobile healthcare system, mHealth, Ecological momentary assessment, Wearable devices

## Abstract

The advancement of wearable/ambulatory technologies has brought a huge change to data collection frameworks in recent decades. Mobile health (mHealth) care platforms, which utilize ambulatory devices to collect naturalistic and often intensively sampled data, produce innovative information of potential clinical relevance. For example, such data can inform clinical study design, recruitment approach, data analysis, and delivery of both “traditional” and novel (e.g., mHealth) interventions. We provide a conceptual overview of how data measured continuously or repeatedly via mobile devices (e.g., smartphone and body sensors) in daily life could be fruitfully used within a mHealth care system. We highlight the potential benefits of integrating ecological momentary assessment (EMA) into mHealth platforms for collecting, processing, and modeling data, and delivering and evaluating novel interventions in everyday life. Although the data obtained from EMA and related approaches may hold great potential benefits for mHealth care system, there are also implementation challenges; we briefly discuss the challenges to integrating EMA into mHealth care system.

## Background

In addition to their primary purposes, advances in mobile communication technology offer great potential to help people become healthier; such technologies can support a variety of public health goals, such as adopting and sustaining a wide range of health behaviors [1]. Technology has long held promise for enhancing the reach of care-services and intervention delivery. For example, people from low income, rural, and aging groups could benefit greatly because technology can facilitate the provision of health services in cases where there are geographic or access barriers (e.g., by delivering care remotely) or cost barriers (by potentially being able to offer services at lower costs due to scale, automation, or other features). Thus, integrating mobile technology into the health care system could potentially have a major impact on enhancing outcomes and reducing care disparities by increasing opportunities for underserved populations to monitor their self-care and better receive healthcare services.

Beyond this, an additional potential benefit of using mobile technology data collection methods is that such methods can collect and provide relatively “real-time” information – such data have the potential to enhance care (e.g., intervention) delivery. For example, ecological momentary assessment (EMA) is a method for acquiring the repeated collection of a person’s momentary experiences in daily life, enabling us to capture the time course of target variables with related factors together in its natural environment [2, 3]. Thus, if mobile/remote monitoring using EMA is available, it becomes possible to deliver care at the moment and location it is needed. A wide array of wearable commercial devices currently also enables useful information related to health status delivering to be automatically transmitted to the individual/patient and/or their caregiver (e.g., by linked smartphone App, by email, web-portal). In addition, unlike care provision that requires direct (often face to face) access to health care experts, these types of systems have the potential to be available all the time. This continuous accessibility could be particularly helpful for handling diseases that may need daily and sometimes even hourly monitoring and adjusting.

Mobile health (mHealth) is a general term for the use of mobile devices and other wireless technology in health care [4]. The broad application of mHealth is to use mobile devices to improve users’ health and well-being by surveillance/analysis of their health status, prevention/treatment support, and chronic disease management/intervention in natural settings. However, despite these potential benefits, the lack of standardized procedures (or even well-developed conceptual models) of how to process, model, and disseminate the data often impede the effective implementation and/or utilization of mHealth systems. Thus, integrating EMA into mHealth care systems means that we use EMA for all these procedures [i.e., data collection, modeling, and interventions; see also Kubiak & Smyth, in press [5]]. Although attempting to address every issue related to mHealth design, implementation, and content is of course well beyond the scope of this article, we attempt herein to outline some potential benefits of the integration of EMA with mobile technologies into mHealth platform and being to address a range of issues in this field. Specifically, the aims are to 1) present benefits of using EMA for a mHealth care platform as a flexible and sustainable infrastructure to advance data collection and model health-related variables, 2) offer ideas on how EMA approaches potentially facilitate the realization of intervention via mHealth care system, and 3) briefly discuss some of the challenges to implementing EMA data on the system.

## Benefits of using EMA on mHealth care systems

The possible capabilities of mHealth care systems largely focus around extending “traditional” health care services from acute care delivery directly from providers in a care setting to enabling remote and ongoing data capture and analysis, by creating a network of communication channels, and by helping individuals keep engaging in their own care in everyday life [6]. However, the current mHealth care systems are generally implemented based on information typically collected by irregular cross-sectional clinical data (e.g., clinical visit, electronic health record [EHR]). Also, recent research in mHealth has often focused on the development of smartphone apps that connect to wearable devices using passive sensing technologies (e.g., activity, sleep, or heart rate monitoring) [7]. Thus, although informative, this type of data collection does not comprehensively evaluate individual’s daily experiences related to health status.

In contrast, EMA approaches can capture more detailed time courses of individual’s psychosocial and subjective experiences and how they relate to other phenomena (e.g., physiological states), albeit at a cost of respondent burden. EMA via smartphone applications can prompt users to report particular behaviors, subjective experiences, and various contextual variables. By using EMA, we can collect detailed information on contextual cognitive and subjective experiences around many of our everyday life behaviors and decisions. For example, reducing smoking is a public health care priority. EMA is helpful to monitor the temporal dynamics of affect, craving, and other experiences (e.g., who the smoker typically smokes with); if such processes show reliable patterns of change (e.g., from just prior to following the smoking behavior) it helps us infer an ecological explanation of the reasons for one’s smoking [8]. Further, these EMA data can be synchronized with other sources of data including passive sensor data (e.g., of physiological systems), environmental features (e.g., ozone levels), and clinical (e.g., pulmonary function) data. Aspects of cardiovascular function coupled with other time-varying psychosocial processes (e.g., affect and craving) can provide unique information regarding smoking behavior [9]. Such data can continue to be “layered” onto; for example, the space-time distribution obtained by global positioning system (GPS) data can provide information where and when smoking behaviors occurred [10] and is possible to add to other (e.g., EMA, sensor data, etc.) data streams. Thus, we see EMA data collection as one important component to extend and enhance the quality of health data as well as effectively monitor and evaluate multi-dimensional health data. It shows great potential for enhancing a mHealth care system because such EMA data may be essential for planning and management of health care [11].

In addition to the data collection benefits, EMA can provide significant development in the modeling of health data, which is thought to be essential to realize the mHealth care systems. A major issue with modeling health data has been the lack of high-resolution data on changes in both predictor and outcome variables in everyday settings [12]. Recent advances in mobile technology have brought enormous opportunities for improving data collection with optimal timescale and appropriate granularity. In addition, EMA allows integrating a broader range of self-reports data (i.e., psychosocial, cognitive, and subjective experiences) simultaneously with other health-related variables often obtained by passive sensing technologies. It thus enables us to comprehensively analyze health-related variables and draw inferences about health status (both overall, for the person, as well as for variation over time within persons). One of the attempts is constructing dynamic computational models (e.g., Cyber-physical systems) which often automatically quantify the multivariate health-related variables and identify the associations (and/or causality) among those variables [12]. For example, understanding (re) lapse behavior is important for managing addiction (e.g., smoking). Lapse behavior does not occur at random, but typically occurs in naturalistic settings and away from a clinic or other health-care setting. As such, ambulatory methods, such as EMA, has the potential to capture this in situ data. Moreover, the rich, often integrative, data streams these methods produce have the potential to be used in novel ways – for example, they can be used to predict and intervene upon behaviors (such as relapse) using dynamic computational models [13]. The models based on such integrated mHealth care systems (e.g., behavioral, physical, social, biological and environmental monitoring in combination with EMA self-reports) may thus have a better chance of quantifying relevant behaviors and the various influences on naturally occurring health behaviors. Further, such data-driven and quantitative model-based approaches can lead to interventions/treatments that are personalized, contextualized, delivered when and where needed in daily life. We turn to this issue below.

## Integrating EMA with interventions via mHealth care system

The mHealth care system has the potential to provide individuals with many kinds of support outside of health facilities at individual level. Mobile and sensor applications can monitor individuals’ health status and gently encourage them to engage in healthy behaviors based on validated dynamic computational models. Conventional ways to intervene target health behaviors, psychological states, or diseases often include sparse and regular schedules across a relatively long-term period (e.g., 3 months) and evaluation of the effects after interventions. Thus, they do not allow to consider when it is most/more effective to deliver interventions [14]. Consequently, it may be difficult to prevent undesirable behaviors and psychological conditions for health via the traditional intervention method, especially for the behaviors and psychological conditions that transpire in natural settings, over relatively short time-frames, and are emergent from the interactions of complex and dynamic psychological, physiological, behavioral, and/or environmental processes.

However, recent approaches to interventions, which have been implemented on mHealth care systems, involve multiple time-varying interventions in a short period (e.g., several times per day) in ecological contexts. For example, the Just-In-Time Adaptive Intervention (JITAI) is an intervention method aiming to deliver appropriate support at the right timing/place according to the contexts that individuals experience at the moment [15]. Advances in EMA data collection and modeling have brought opportunities for implementing these JITAI interventions in personalized medicine on mHealth care systems.

With JITAI type of interventions, the questions of when and where to implement them are central to address and researchers often use predefined decision rules. For example, in deciding to provide interventions, for the first step, a health-related behavior (e.g., sleep, eating behavior, or physical activity) or a psychological state (e.g., depression and stress level) may be selected as a target of intervention. And then, as a way to improve/manage the target behavior or psychological state, intervention can be triggered based on predefined decision rules. These decision rules can be based on dynamic computational models derived from EMA data according to the target health-related variables [16]. Specifically, we may be able to compute the *moment* when a health indicator suggests risk (e.g., undesirable behaviors and psychological conditions for health) to define an appropriate moment to deliver an intervention [17]; essentially, there is emerging evidence that providing intervention support at moments of risk provides benefit beyond standard (e.g. in-person) delivery and simple reminder-based systems [18]. The computations can be enhanced through sophisticated processing and modeling by utilizing person-specific estimates (e.g., each individual functioning as their own comparison) using intensive data (e.g., EMA, passively collected), and by allowing estimates and projections (e.g., calculations of moments of risk) to update in real time, over time, to more accurately reflect the intra-individual dynamics of health processes and health behavior enactment [19]. Such information also holds potential to continuously improve intervention timing, dose, delivery, etc., by adapting to individuals’ dynamic experiences – this might include, but is not limited to, burden (e.g., number/dose of interventions in some unit time), social environmental factors, availability for intervention, location, and so forth.

Beyond the timing of intervention, its contents and modalities are also important elements to consider. There are many approaches to constructing intervention elements to enhance health-related processes. For example, we can use active supports via useful information related to health or mere feedback on individual’s health status for different situations. Also, these intervention types can be delivered using various intervention means such as messages, interactive messages, phone calls, images, audio, video, and so forth. Further, we see great potential to optimize intervention design and delivery by careful consideration of the intervention target (i.e., which behavior or psychological state is targeted) and content (i.e., how and how fast/long/much you intervene).

“DietAlert” developed by Goldstein and colleagues [13] is an application that aims to help overweight and obese individuals avoid dietary lapses after a weight control diet. Participants are asked to record data about lapses from their diets and a range of potential triggers using EMA. Dynamic computational modeling is used to generate models that estimate the level of upcoming risk for lapse of eating behavior based on current context. When an EMA recording from a participant meets predefined decision rules that indicate a high-risk moment, a series of brief intervention elements (e.g., brief text message) are delivered to help prevent lapses. Further, the effect of any intervention efforts can be immediately evaluated (e.g., by examining the following EMA, whether a lapse actually occurred or not, etc.) and the predictive learning algorithm can be adjusted. Each of these, and other, intervention processes have the potential to be informed by EMA data collection with ambulatory technologies as well as models derived from the data via mHealth care platforms.

## Implementation challenges for mHeath care systems

Despite the great potential of using EMA data on mHealth care systems, there are many challenges to the development, implementation, and utilization of such systems. These include, but are certainly not limited to, data quality and valid data sampling rate, model development, and model validation in intervention studies. Despite the proliferation of assessment systems (both in the marketplace and for research purposes), as well as for wearable sensor devices (e.g., low cost “fitness trackers”), there is remarkably little evidence regarding their reliability and validity. If we wish to integrate such approaches into clinical care, it is essential to ensure sufficient data quality by validating any new assessment tool and establishing data capture standards before applying the tool in mHealth care systems. Many validation studies have been conducted for wearable devices and smartphone applications. A review study conducted a meta-analysis using 60 studies validating energy expenditure estimates from activity monitors against criterion measurements (i.e., indirect calorimetry, room calorimeters, and doubly labelled water) [20]. This study showed that the accuracy of energy expenditure estimates from 40 activity monitors differs depending on activity types (see Supplementary materials 5 in the study for characteristics of the activity monitors and the measurement error for each activity monitor). A study examined the accuracy of smartphone applications and wearable devices against directly observed actual step counts [21]. The validity of heart rate monitors using a wrist-worn or chest strap for electrocardiography (ECG) detection was examined by comparing with multi-lead ambulatory ECG which considered as the gold standard [22, 23]. The feasibility of a remote patient monitoring system using the knee sleeve and smartphone in total knee arthroplasty was validated/established in terms of the continuous (uninterrupted) data collection and patients’ engagement of technology [24].

In addition to the overall accuracy of the tools, it is necessary to determine which sampling rate is appropriate for integrating different data (e.g., EMA with clinical and passive sensor data). Here it is important to be careful about the burden of high frequencies of self-reported EMA. For example, understanding how frequently to monitor specific behaviors (e.g., eating vs. sleeping) will contribute to the understanding of contextual effects on the behaviors. For eating, we might readily speculate that the sampling rate should be greater than once a day to cover most eating behaviors during a day, whereas for sleeping that intensity may be less well suited (likely occurring nightly, notwithstanding napping behavior). Intensive sampling, however, is extremely burdensome for participants, for example, if they need to manually input eating records; this makes it difficult to keep participants engaged and adherent over lengthy periods. As such, it is important to empirically evaluate and consider a careful tradeoff between adequate sampling rates and participant burden for EMA data capture. Passive sensing data can, in most cases, be continuously measured without much respondent burden (e.g., infrequent data management, battery charging, etc.) but challenges with data integration (e.g., over what time-frame to aggregate and merge with EMA) exist. As one example in this area of enquiry, a study systematically examined the associations between depressive mood (EMA) and physical activity (passive sensor) using a variety of aggregation time frames for physical activity; in brief, their findings suggested that one-hour physical activity epochs are appropriate to investigate the association with depressive mood [25]. Also, a study represented the new research platform “Physiqual,” which integrates physiological sensor data with EMA such as self-reports data [26]. This study comprehensively discusses how to gather and combine wearable sensor technology with EMA, which may give new insight regarding the question of adequate sampling rate for integrating multiple data streams. More research and development may be needed to set up the analogous sampling rates for other health-related variables.

Along with assuring the sufficient data quality and valid sampling rate, sophisticated modeling of the data is an important element for mHealth care systems. Although many dynamic computational models are applied to study the complexity of health status, it is necessary to ensure that the proper types of modeling are performed without bias by validating the models in intervention studies [27]. Here we introduce some literature which provide a useful information for sophisticated modeling regarding the management/intervention of health behaviors [28, 29] and detailed techniques for designing a validation study to test the effect of mHealth interventions [30, 31]. It is also useful to establish the implications of causality between health-related variables by combining data-driven approaches with multiple dynamic computational models estimated from multidimensional data (e.g., time courses of psychological, behavioral, biological, physiological, social, and environmental variables) obtained by EMA and other (e.g., passive sensors) data streams [32]. In other words, the models ideally should go beyond a specific area of health and integrate models of multiple health processes. This approach will facilitate our understanding of factors that may influence health-related variables.

Ultimately, our hope is that these near-continuous multi-method monitoring systems, including sophisticated EMA data collection, and dynamic modeling approaches will help developing successful mHealth care systems that support optimized, efficient, and high-reach/scalable interventions. However, ensuring safety of the user (especially patients) and efficacy of the systems, particularly interventions, are each essential [12]. The effect of such mHealth interventions should thus be carefully considered in a way to validate them to maximize their safety and efficacy. In addition to “traditional” developmental pathways and evaluations (e.g., randomized controlled trials), there are emerging methods for testing, optimizing, and evaluating novel intervention approaches [33]. It is likely that are careful balance of these approaches will help this field move forward in a safe and efficient manner.

## Conclusion

Health care experts have advocated the use of mobile technologies to improve the fragmented health care system for decades [1]. One (of many) potential ways to enhance this system and improve care is through the integration of EMA-derived patient reports into broader mHealth care systems. EMA using mobile data collection is already an essential research tool in many fields of research and clinical settings; additionally, wearable body sensors allow for large quantities of data to be collected automatically, and can be done in conjunction with EMA reporting. This brings opportunities for integrating such data collection into broader care systems, but also opens up new data processing methods for modeling of predictors and outcome variables related to health and well-being. In addition, the dynamic computational models constructed by EMA data allow to develop novel interventions (e.g., JITAI) and test its effects, leading to a successful implementation of mHealth care systems outside of the traditional points of contact in care systems (e.g., clinic visits) [34]. Overall, we are optimistic that the models developed with temporally dense, multidimensional/integrated data can be used to inform an evidence-based, safe, and effective health-care system and process that enhances the capacity for people to live healthier lives.

## Data Availability

Not applicable.

## Abbreviations

EMA: Ecological Momentary Assessment
JITAI: Just-In-Time Adaptive Intervention
mHealth: Mobile Health
